# Risk assessment of nanomaterials in cosmetics: a European union perspective

**DOI:** 10.1007/s00204-012-0944-x

**Published:** 2012-10-02

**Authors:** Frank Henkler, Tewes Tralau, Jutta Tentschert, Carsten Kneuer, Andrea Haase, Thomas Platzek, Andreas Luch, Mario E. Götz

**Affiliations:** German Federal Institute for Risk Assessment (BfR), Berlin, Germany

## Abstract

In Europe, the data requirements for the hazard and exposure characterisation of chemicals are defined according to the REACH regulation and its guidance on information requirements and chemical safety assessment (Regulation (EC) No 1907/2006 of the European Parliament and of the Council of 18 December 2006 concerning the Registration, Evaluation, Authorisation and Restriction of Chemicals (REACH), and its guidance documents; available at: http://eur-lex.europa.eu/LexUriServ/LexUriServ.do?uri=OJ:L:2006:396:0001:0849:EN:PDF; and at: http://guidance.echa.europa.eu/docs/guidance_document/information_requirements_en.htm). This is the basis for any related risk assessment. The standard reference for the testing of cosmetic ingredients is the SCCP’s ‘Notes of Guidance for the Testing of Cosmetic Ingredients and their Safety Evaluation’ (The SCCP’s Notes of Guidance for the testing of cosmetic ingredients and their safety evaluation (2006); available at: http://ec.europa.eu/health/ph_risk/committees/04_sccp/docs/sccp_o_03j.pdf), which refers to the OECD guidelines for the testing of chemicals (The OECD Guidelines for the Testing of Chemicals as a collection of the most relevant internationally agreed testing methods used by government, industry and independent laboratories to assess the safety of chemical products; available at: http://www.oecd.org/topic/0,2686,en_2649_34377_1_1_1_1_37407,00.html). According to the cosmetics directive [76/768/EEC], compounds that are classified as mutagenic, carcinogenic or toxic to reproduction are banned for the use in cosmetic products. Since December 2010, the respective labelling is based on the rules of regulation (EC) No. 1272/2008 (Regulation (EC) No 1272/2008 of the European Parliament and of the Council of 16 December 2008 on classification, labelling and packaging of substances and mixtures, amending and repealing Directives 67/548/EEC and 1999/45/EC, and amending Regulation (EC) No 1907/2006, Official Journal L 353, 31/12/2008, pages 1–1355; available at: http://eur-lex.europa.eu/LexUriServ/LexUriServ.do?uri=OJ:L:2008:353:0001:1355:en:PDF) on classification, labelling and packaging of substances and mixtures (CLP). There is no further impact from the CLP regulation on cosmetic products, because regulation (EC) No. 1223/2009 on cosmetic products defines its own labelling rules (Regulation (EC) No 1223/2009 of the European Parliament and of the Council of 30 November 2009 on cosmetic products; available at: http://eur-lex.europa.eu/LexUriServ/LexUriServ.do?uri=OJ:L:2009:342:0059:0209:en:PDF). Special notification procedures are mandatory for preservatives, colourants and UV-filters where a safety approval from the European ‘Scientific Committee on Consumer Safety’ (SCCS) is needed prior to marketing. The risk assessment of nanomaterials in consumer products still poses a significant challenge as highlighted by the example of UV-filters in sunscreens since nanomaterials cannot be classified as a homogenous group of chemicals but still need to be addressed in risk characterisation on a case by case basis.

## Nanomaterials in cosmetic products—the new European ‘Cosmetic Regulation’

Existing regulations and associated guidance documents can, in principle, be adapted to nanomaterials (NM). However, it is often difficult to perform a sound hazard and exposure assessment, since the current legal framework does not account for the complex physico-chemical properties of manufactured NM or the respective analytical requirements. Likewise, there is a frequent lack of suitable toxicological methods. Nevertheless, the European Parliament must find feasible solutions that guarantee consumer protection at the highest standards possible. Rules need to be flexible enough to allow for a timely adaptation of current laws to new scientific knowledge as well as to respond to profound concerns raised by official institutions in the EU member states or by competent third parties.

The new European ‘Cosmetic Regulation’ was adopted (1223/2009) in 2009 and will become operative by 11 July 2013. Article 2 (k) of this regulation provides the first official definition of NM within the European legislative framework. In this context, NM is defined as an insoluble or biopersistent and intentionally manufactured material with one or more external dimensions, or an internal structure, on the scale from 1 to 100 nm.

**Fig. 1:**
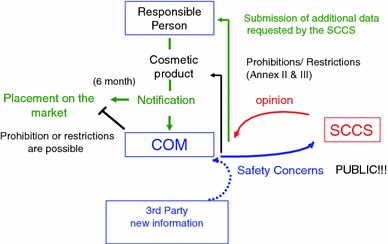
Notification process for cosmetics that contain NM according to the new EU Cosmetic Regulation 1223/2009

The ‘Cosmetics Regulation’ is the first act in Europe that explicitly considers the putative risks from NM on consumers. Its article 16 is solely dedicated to NM. Each cosmetic product requires a designated ‘responsible person’ for a placement on the European market. This representative is requested to notify the European Commission 6 months in advance before any cosmetics containing NM are marketed. Required specifications for NM include particle sizes, used raw materials and information on product impurities. In addition, a toxicological profile needs to be provided that covers all relevant endpoints, especially skin and eye irritation, as well as skin sensitisation (Annex I, 6). The European Commission (COM) may request an opinion from the ‘Scientific Committee on Consumer Safety’ (SCCS) in case of safety concerns about the respective product notification or the particular NM used in the cosmetic product. This opinion has to be provided within 6 months and may ask for additional data, recommend a restricted application, or even propose the ban of the corresponding substance. Opinions by the SCCS will be published by COM. Safety concerns initiating a further or new evaluation process can also be raised after a substance has been placed on the market. Reasons for such action include new scientific knowledge as well as novel data on substance exposure or toxicology as provided by third parties (i.e. institutions of the member states). This can also lead to further requirements for NM in general. The notification process for NM, as outlined in article 16, does not apply for materials that are intended to be used as preservatives, colourants and UV-filters, as these preparations still need to be approved by inclusion into annexes IV–VI. Moreover, COM is responsible for market and safety surveillance measures and will provide an up-to-date catalogue of NM used in cosmetics (Fig. 1).

## Analytical challenges specific for nanomaterials

Nanomaterials are at the borderline of easily soluble small molecules and large, hardly soluble materials and dusts. As a consequence, they cannot be routinely detected by conventional microscopy. Exposure assessment and dose metrics have to be specifically addressed by analytical tools that enable tracing and quantification of substances at the nanoscale. Such techniques are very costly and differ from conventional analytics. Further, their applicability strongly depends on the analysed matrix. Hence, there are strong efforts to develop analytical and toxicological methods fit for the nano-world to enable valid dose description and risk characterisation.

Analytical measurements at the nanoscale have to meet the requirements of measuring an array of physico-chemical parameters, which fully characterise the respective NM in a given matrix. Inductively coupled plasma-mass spectrometry (ICP-MS) allows element concentrations to be measured, while transmission electron microscopy (TEM) can be used to determine the size and the shape of the material used. However, subsequent characterisation requires a whole portfolio of detailed analytical techniques. Besides two different analytical tools necessary to determine the size of the NM under consideration, it is central to conduct the measurements in the most meaningful environment (matrix). It is well known that NM can change their chemical surface and their reactivity depending on the particular medium they are dispersed in. As a consequence, one has to establish not only the appropriate analytical tools but also the right sampling time and the correct analytical sequence. Otherwise, results might be biased thus leaving any further exposure assessments in doubt. In this respect, a good understanding of the ‘history’ of the specific NM may prove helpful. Examples are the knowledge on the way the NM is synthesised and manufactured, how it is subsequently incorporated into other materials and whether there are additives or catalysts applied. The resulting changes in the surface chemistry and reactivity will not only significantly affect the physico-chemical characteristics of NM but also may influence its toxicological properties. A combination of well-established technologies could be considered for chemical analysis of surface modifications. The characterisation of the coating or capping of NM can be established by thermogravimetry in combination with GC–MS, while tandem LC–MS allows for identification and quantification of the chemical coatings and stabilisers used.

## Data requirements for safety assessment

From a regulatory perspective, there are two other major issues that distinguish the risk assessment of NM from that of conventional chemicals. Notably, it is usually unknown whether the nano-claims of manufacturers are trustworthy and substantiated for individual products. In Germany, there are presently no requirements for the documentation or monitoring of nano-properties in products that are supposed to contain NM. In addition, our knowledge about specific toxicological features of NM is still insufficient.

It has been shown that some types of NM can elicit proinflammatory effects in rats upon inhalation. This data prompted increased research efforts on hazard identification in the fields of occupational exposure and health safety. Meanwhile, NM are widely used as UV-filters in sunscreens and recent developments point to a much wider functional spectrum in cosmetics, including moisturising cremes, so-called anti-ageing products, preservatives and as carrier systems for macromolecules such as collagen.

For cosmetic ingredients, the basics of risk characterisation are laid down in the SCCP’s ‘Notes of Guidance for the Testing of Cosmetic Ingredients and Their Safety Evaluation’, which has not yet been adapted for the risk assessment of NM. This is mainly due to the fact that an adaption of the REACH guidance documents is likewise pending, as is a European definition for NM under REACH. The reason is that the implementation of nano-specific regulation relies on systematic concepts of risk assessment trustfully applicable in the various fields of NM application (i.e. as a chemical substance, or part of a consumer product). Such concepts have yet to be established and existing analytical and toxicological methods need to be adapted accordingly.

At present, regulators become challenged by a wide range of NM testing protocols that mainly differ in test item preparation techniques and characterisation tools. Further, there are discrepancies in dose metrics and a lack of performance standards. In these respects, all in vivo and in vitro methods proposed or considered to be applied in NM testing need to undergo a critical reappraisal and adjustment if necessary.

Based on these precautionary notes, it appears reasonable to assume that the data requirements for chemical substances and cosmetic ingredients will be adapted in the near future. A scientifically driven risk characterisation will require a basic dataset of physico-chemical characteristics of the corresponding NM as a pure substance as well as in the respective product formula as marketed.

## Global efforts on method adaptation

In a global effort, the OECD as well as ISO is struggling very hard to generate guidance standards for hazard and exposure identification and quantification.

The OECD currently runs a stewardship programme for the testing of a selected set of different manufactured NM and alternate NM with different surface modifications (OECD 2010). In addition, the applicability of existing OECD test guidelines for the safety assessment of NM is under review.

Main safety concerns on NM are related to size, surface area and surface reactivity. The latter comprises surface chemistry and composition (coatings) as well as redox and other catalytical reactions. Systematic investigation of the nano–bio interface is much discussed. However, respective studies are just starting to emerge. In a regulator’s view, these concerns have to be translated into the formulation of criteria on data requirements for future guidance notes. These notes are then to be used for a safety assessment that accounts for the whole life cycle of NM. A mandate to amend the REACH guidance accordingly asks for the completion of so-called REACH implementation projects by 2013.

Nevertheless, data requirements may be well different, depending on the intended use and the possible sites of contact or the expected levels of exposure. Consequently, different testing strategies may evolve depending on the possible exposure scenarios identified.

## Open issues in risk assessment

Authorities and scientific committees have independently concluded that the existing approach for chemical risk assessment should also be applicable to NM (e.g. SCENIHR 2007; EFSA 2009; FAO/WHO 2009). This approach is also known as the ‘risk assessment paradigm’ and includes hazard identification, hazard characterisation (dose–response analysis), exposure assessment and final risk characterisation as major steps.

However, the application of this risk assessment paradigm to NM raises a number of issues, which are more or less critical for the outcome of the assessment. One problem is the consideration under which conditions the existing data for the respective material can be used, as such data can comprise the bulk, the molecular, or similar nanoforms of a particular material. Further, one has to be aware about the degree of added uncertainty. In addition, a number of (default) factors routinely applied in risk assessment, such as those for inter- and intraspecies variability or time extrapolation (e.g. subchronic to chronic) were developed based on data for conventional chemicals. It is currently unknown whether these factors can be confirmed also for NM. Hence, this should be noted as a source of additional uncertainty while applying established defaults in the absence of alternatives. These and other open points in the risk assessment process for NM are currently addressed by the OECD WPMN and other groups.

## Data requirements for cosmetic products containing nanomaterials

With regard to the aforementioned risk assessment of NM data on the dermal contact, skin penetration and skin absorption are of special importance. NM that do not reach the living cells of the epidermis may be regarded less harmful than others as the latter may potentially be absorbed into the blood stream, thus allowing their distribution to secondary target organs.

As indicated earlier, the matrix or mixtures in which NM are applied may critically change its physico-chemical characteristics in the final product and coatings or impurities may be released. This may render the material more or even less toxic. Therefore, size distribution, surface chemistry and reactivity towards potential biomolecular targets (e.g. in skin or lung) must be investigated. Hence, such a characterisation should regard the possible solvent conditions in situ and include dispersions in water, sweat stimulants, lung surfactant or buccal mucosa. Likewise, the properties of NM may change also during storage and handling.

However, the main concerns about NM in cosmetic products are the possible translocation to viable skin cells, its genotoxic, proinflammatory or sensitising activities and the influence of UV light on these parameters.

## The case of nanoparticulate ZnO as UV-filter used in sunscreens

Micronised ZnO is widely used in sunscreens. As particulate formulation, it reflects, absorbs and refracts UV-radiation. The difficulties to evaluate putative risks to humans illustrate general problems in the risk assessment and classification of NM. The potential risks of NM had been known for several years, but the requested studies to address these concerns have still not been provided. In 2003, the ‘Scientific Committee on Cosmetic and Non-Food Products’ (SCCNFP) tried to clarify the putative risks of the use of ZnO in sunscreens and published an opinion on behalf of COM (SCCNFP/0649/03). This report discussed the general toxicity of ZnO in detail and concluded that Zn^2+^ ions can be considered non-toxic. The SCCNFP further evaluated toxicological tests that had been carried out with micronised ZnO. Micronised ZnO consists of core particles which can display diameters below 100 nm and thus need to be classified as NM. These particles are usually coated with inorganic or organic (dimethicone) compounds, leading to an increased dimension of up to 200 nm. Although most toxicological data available to the SCCNFP did not point to significant risks, in vitro mammalian chromosomal aberration tests revealed photoclastogenic and possibly photoaneugenic effects. According to the SCCNFP, these photogenotoxic effects need to be further investigated in vivo. A complete toxicological dossier for micronised ZnO was requested, including a report on possible pathways for the cutaneous penetration and systemic exposure. These requests were confirmed by the SCCNFP in 2005 (SCCNFP/0932/05), concluding that the safety of micronised ZnO remains uncertain, because essential information was still not provided. However, in 2009, the ‘Scientific Committee on Consumer Products’ (SCCP) confirmed that the use of ZnO in its non-nano form (micronised, pigment grade) is considered safe on the basis of the initial dossier (SCCNFP/0649/03). Previous concerns were no longer regarded as relevant due the absence of dermal penetration (SCCP/1215/09). Further, no indications had been found for genotoxic activities of micronised ZnO in vivo so far (Schilling et al. 2010).

This example illustrates several problems. Firstly, it is not yet conclusively clarified whether nano-sized ZnO can penetrate into human skin. Notably, particles of diameters below 12 nm have been demonstrated to penetrate into intact skin (Ryman-Rasmussen et al. 2006). Hence, this option should not be ruled out for even slightly larger particles without a detailed experimental analysis. Initial investigations on dermal penetration of ZnO and titanium dioxide (TiO_2_) particles were carried out by Gamer et al. (2006) on dermatomed porcine skin. After a 24-h exposure, ZnO particles with an average size of 80 nm were quantitatively recovered from the skin surface, indicating that no penetration occurred. However, traces of labelled ^68^Zn were found in the blood and urine of human volunteers following skin exposure to particles of 19 and 100 nm, respectively. Nevertheless, the results further showed that only 1/1,000 of totally applied ZnO was found in blood over 5 days. This suggests that the overwhelming proportion of Zn was not absorbed (Gulson et al. 2010). So far, it is not yet clarified whether the ^68^Zn isotope was absorbed in its nanoparticulate form or as Zn^2+^ ions, which might have been released during exposure. Another study using multiphoton tomography showed that 26–30 nm sized ZnO particles coated with polymethylsilsesquioxane did not migrate into the living layers of human skin (Roberts et al. 2008).

A second problem relates to the general transferability of the previous conclusions to nano-sized ZnO, because micronised ZnO preparations are often not well-defined but may contain nano-sized ZnO to a great extent. In respect to requested further tests, a thorough physico-chemical characterisation of the test item in each test system as proposed by the OECD WPMN is required. Hazard identification of the differently coated nano-sized ZnO as marketed should focus on translocation through portals of entry and target organ toxicity at the site of contact. Analytically, data on the kinetics of ZnO dissolution in skin, blood and even secondary organs following body distribution should become available for each formulation of ZnO. The stability of coatings with regard to the intended use has to be analytically addressed as well. Beyond these novel aspects, the SCCP did also summarise central points that still need to be improved or clarified (SCCP, 2007). Further uncertainties relate to an exposure to nanoscaled ZnO particles via inhalation as it can occur during production and transport (Osmond and McCall 2010). However, gas driven aerosol spray applications are not permitted under current legislation.

Although the BfR does not recognise any considerable health risk for consumers who use products that contain micronised ZnO, the final recommendation about its use as a UV-filter in cosmetic products should be issued by the SCCS. Such a decision should be based on the data that were requested in 2003. The required information ought to be available soon, given that a corresponding dossier for the evaluation under REACH has already been submitted.

Another NM used for its UV-protecting properties in sunscreens is TiO_2_. The issue of translocation of nano-TiO_2_ was addressed by the German research project NanoDerm. It was found that particles with a diameter of more than 20 nm can be considered safe as they do not reach viable cells in the epidermis. Further, the toxicological profile of TiO_2_ did not raise any safety concerns regarding its application (SCCNFP/0005/98) to non-flexed and unburned human skin. Consequently, the exclusion of particles of less than 20 nm in the production process would improve product safety for this particular compound.

## Addressing consumer concerns

Consumer trust in products can be enhanced by transparent data communication. Therefore, manufacturers are urged to provide reliable data on the effectiveness of sun protection and the absence of any NM translocation into the blood stream. Based on the currently available data, for NM routinely used in sunscreens, it seems unlikely that particles with a hydrodynamic diameter larger than 20 nm may reach viable skin cells, thus making translocation into systemic circulation implausible.
